# Rumen and cecum microbial dynamics following narasin inclusion in Nellore cattle diets

**DOI:** 10.3389/fmicb.2026.1645979

**Published:** 2026-02-13

**Authors:** Johnny M. Souza, Leandro A. F. Silva, Daniel M. Casali, Janaina C. S. M. Souza, Luke A. Wolfe, Joseph H. Skarlupka, Ibrahim Zuniga-Chaves, Andrew J. Steinberger, Courtney L. Deblois, Andrew J. Scheftgen, Ana L. J. Lelis, Tiago Leiva, Murilo Chuba Rodrigues, José Paulo Roman Barroso, Pedro Veloso Facuri Lasmar, Garret Suen, Danilo D. Millen

**Affiliations:** 1 Department of Animal Science, São Paulo State University (UNESP), School of Agricultural and Veterinarian Sciences, Jaboticabal, São Paulo, Brazil; 2 Department of Animal Science, São Paulo State University (UNESP), School of Veterinary Medicine and Animal Science, Botucatu, São Paulo, Brazil; 3 Department of Nutrition and Animal Production, University of São Paulo (USP), Pirassununga, São Paulo, Brazil; 4 Department of Bacteriology, University of Wisconsin-Madison, Madison, WI, United States; 5 Elanco Animal Health, São Paulo, SP, Brazil

**Keywords:** 16S rRNA gene, beef cattle, feedlot, ruminants, sequencing

## Abstract

This study investigated the effects of narasin supplementation on the ruminal and cecal bacterial communities of feedlot Nellore cattle. We hypothesized that narasin would selectively modulate microbial populations in distinct gastrointestinal compartments without causing broad-scale disruption of overall community diversity. Sixty-four Nellore bulls (393 ± 24 kg) were assigned to a completely randomized block design and fed finishing diets containing either 0 or 20 ppm of narasin for 112 days. Rumen and cecal contents were collected at slaughter and analyzed using 16S rRNA gene sequencing to characterize bacterial community structure and composition. Overall, the rumen exhibited greater bacterial diversity and richness than the cecum, regardless of dietary treatment. Narasin supplementation did not affect Shannon diversity in either the rumen (*p* = 0.182) or the cecum (*p* = 0.298); however, Chao richness was reduced in the rumen of narasin-fed cattle (*p* = 0.028). Beta-diversity analyses based on Bray–Curtis and Jaccard dissimilarities revealed no significant differences in overall community structure between treatments in either compartment (*p* > 0.198). At the phylum level, narasin supplementation was associated with a reduction in *Firmicutes* and a concomitant increase in *Bacteroidetes* in the rumen. In contrast, *Firmicutes* predominated in the cecum, and narasin significantly increased the relative abundance of this phylum, particularly members of the order *Clostridiales* (*p* = 0.05). In conclusion, narasin exerts selective effects on specific bacterial populations rather than inducing widespread shifts in microbial diversity. These results provide novel insights into how narasin modulates microbial ecology in both the rumen and the understudied cecum, highlighting compartment-specific responses that may contribute to improved feed efficiency in beef cattle.

## 1 Introduction

Feedlot diets that are high in energy, particularly those rich in starch, often lead to an increase in short-chain fatty acids (SCFA) in the rumen of cattle. This can result in temporary drops in ruminal pH, which may cause metabolic issues such as ruminal acidosis, subsequently affecting cattle feed intake (Owens et al., 1998). Ruminal acidosis is one of the primary health challenges faced by dairy and feedlot cattle (Silvestre and Millen, 2021; Golder and Lean, 2024). Moreover, numerous studies have focused on preventing or mitigating acidosis to enhance the performance and efficiency of feedlot cattle (Owens et al., 1998; Brown et al., 2006).

Nellore cattle (*Bos indicus*) represent the predominant beef cattle breed in Brazil and account for a substantial proportion of beef production in tropical and subtropical regions worldwide. Compared with *Bos taurus* breeds, Nellore cattle exhibit distinct physiological and metabolic characteristics, including differences in feed efficiency, ruminal fermentation patterns, and host–microbiome interactions. Previous studies have demonstrated that both host genetics and breed type can influence ruminal microbial community composition and function, highlighting the importance of considering zebu cattle as a distinct biological model in microbiome research (Henderson et al., 2015). Despite their global relevance, most rumen microbiome studies have focused on taurine cattle, leaving important knowledge gaps regarding the microbial ecology of Nellore cattle under intensive feedlot conditions.

Feed additives, such as ionophores, have been investigated worldwide over the past couple of decades to control ruminal acidification and enhance the performance of feedlot cattle (Silvestre et al., 2023). The primary mechanism of most ionophores is to regulate the growth of gram-positive rumen bacteria, including those that produce lactate in the rumen (Nagaraja and Titgemeyer, 2007). Ionophores alter transmembrane ion gradients in susceptible microorganisms, resulting in shifts in microbial activity and fermentation end-products, such as increased propionate production and improved feed efficiency (Russell and Strobel, 1989; Russell and Houlihan, 2003).

Narasin is one of the ionophores that has been researched for its ability to alter rumen fermentation dynamics (Miszura et al., 2018), resulting in changes in plasma metabolites by increasing glucose levels (Sardinha et al., 2020) and reducing urea concentration (Polizel et al., 2020), which leads to positive results in animal performance (Silva et al., 2015; Polizel et al., 2020). In this context, Limede et al. (2021) reported an increase in ruminal propionate concentration, a reduction in the ruminal acetate-to-propionate ratio, and an increase in total SCFA concentration in response to the inclusion of narasin in the diet of Nellore cattle. Concerning performance, the same authors found that narasin enhanced dry matter intake, average daily gain, and final live weight compared to the control treatment, demonstrating the potential of this ionophore for animal health performance. Although several studies have documented the effects of narasin and other ionophores on ruminal fermentation parameters, information regarding their direct impact on ruminal microbial community structure remains limited and is largely inferred from studies with monensin (Marques and Cooke, 2021). Moreover, the potential effects of narasin on the cecal microbiota—a key site for post-ruminal fermentation—remain largely unexplored, despite evidence that microbial communities differ markedly along the gastrointestinal tract (Wang et al., 2017).

However, previous studies assessing the inclusion of narasin in cattle diets did not examine its effects on bacterial communities in the rumen and cecum. Clarifying narasin’s mode of action is essential to support the reasons for improved performance in cattle consuming this feed additive. Therefore, this study aims to identify bacterial communities through 16S rRNA gene sequencing in the rumen and cecum of Nellore cattle finished in a feedlot on diets containing narasin.

Given the functional differences between the rumen and the cecum and the limited information regarding the effects of narasin on microbial communities across gastrointestinal compartments, a comprehensive evaluation of both sites is warranted. Previous studies have shown that microbial composition and interactions vary substantially along the gastrointestinal tract, reflecting differences in substrate availability and fermentation dynamics (Wang et al., 2017; Malmuthuge and Guan, 2017). We hypothesized that narasin would exert selective pressure on specific bacterial taxa in both compartments, leading to compartment-specific shifts in microbial composition without causing broad-scale disruption of overall community diversity. Therefore, this study aimed to characterize the ruminal and cecal bacterial communities of feedlot Nellore cattle supplemented with narasin using 16S rRNA gene sequencing.

## 2 Material and methods

### 2.1 Animals and experimental design

Animal care and handling procedures used in this experiment adhered to the guidelines of the Animal Use Ethics Committee (CEUA) and were approved by the Ethics Committee on Animal Use of São Paulo State University (UNESP), Dracena campus, Brazil (Protocol CEUA 002/2022).

This study was conducted at the experimental feedlot of the Faculty of Agricultural and Technological Sciences at UNESP, Dracena campus, Brazil. Sixty-four Nellore bulls (393 ± 24 kg) were utilized and randomly assigned to 16 pens (*n* = 4 animals per pen) in a completely randomized block design, with initial live weight serving as the blocking criterion. The study lasted 112 days, after which all animals were slaughtered in a commercial setting slaughterhouse.

The animals were pre-adapted to the facility and were fed Tifton hay for 7 days to standardize the ruminal microbiota population. Subsequently, the animals were randomly allocated within each block to one of the following treatments: 0 ppm (Control) or 20 ppm of Narasin (Elanco Animal Health, São Paulo, SP, Brazil). The additive was provided according to the manufacturer’s instructions. Narasin supplementation was initiated at the beginning of the adaptation period and was maintained continuously throughout the entire experimental period, including the finishing phase, until slaughter.

### 2.2 Management, feeding, and animal care

Upon arrival, the animals were weighed, treated with anthelmintics, and vaccinated against viral and bacterial diseases, including rotavirus, coronavirus, tetanus, botulism, and seven types of Clostridium spp. (Cattlemaster, Pfizer Animal Health). Animals were housed in partially concreted pens, with a stocking density of four animals per pen (18 m^2^ and 1.5 m of linear feed bunk per animal), with unrestricted access to water via automatic drinkers.

Feeding occurred three times a day: at 08:00 (35% of the total), 11:00 (20% of the total), and 16:00 (45% of the total). The study included a 14-day adaptation period following a step-up protocol with three adaptation diets: adaptation 1 (75% concentrate) for 5 days, adaptation 2 (79% concentrate) for 4 days, and adaptation 3 (83% concentrate) for 5 days. The finishing diet comprised 87% concentrate ingredients (Table 1). Experimental diets were formulated according to the Large Ruminant Nutrition System (LRNS; Fox et al., 2004), level 2. Diets were provided *ad libitum* and adjusted daily based on feed bunk refusals, which were maintained at 5% of the offered amount.

**Table 1:** Experimental diets provided during the finishing phase for feedlot Nellore cattle.

Diets^1^	A1	A2	A3	T
Concentrate level (%)	75.00	79.00	83.00	87.00
Ingredients (% DM)				
Peanut Hull	10.00	10.00	10.00	10.00
Hay	15.00	11.00	7.00	3.00
Ground corn	51.00	56.00	61.00	66.00
Soybean meal	10.00	8.00	6.00	4.00
Cottonseed meal	11.00	12.00	13.00	14.00
Urea	0.50	0.50	0.50	0.50
Premix^2^	2.50	2.50	2.50	2.50
Nutritional content				
Dry matter (DM) (%)	89.00	84.00	84.00	84.00
Total digestible nutrients (% DM)	69.00	71.00	73.00	75.00
Crude protein (% DM)	14.70	14.70	14.60	14.70
Neutral detergent fiber (% DM)	33.20	30.20	27.20	24.10
Non-fibrous carbohydrates (% DM)	46.00	49.00	52.00	55.00
Ether extract (% DM)	2.80	2.90	3.00	3.00
peNDF	23.00	20.00	17.00	13.00
NEL (Mcal/kg DM)	1.01	1.05	1.11	1.17
Ca (% DM)	0.57	0.55	0.54	0.56
P (% DM)	0.39	0.40	0.41	0.42

1 A1, adaptation diet 1; A2, adaptation diet 2; A3, adaptation diet 3; T, finishing diet.

2 Composition per kg of dry matter: Calcium 160 g, Phosphorus 22 g, Sodium 70 g, Potassium 40 g, Magnesium 35 g, Sulfur 25 g, Cobalt 30 mg, Copper 450 mg, Iodine 25 mg, Manganese 850 mg, Selenium 5 mg, Zinc 1,350 mg, Chromium 15 mg, Vitamin A 60,000 IU, Vitamin D 8,000 IU, Vitamin E 480 IU The additive was added to the premix to make a total of 20 ppm of Narasin.

After 112 days in the feedlot, animals were slaughtered at a commercial slaughterhouse, reaching an average final live weight of approximately 520 kg. The contents of the rumen and cecum were collected post-slaughter from all animals and stored at −80 °C for subsequent bacterial community analysis.

### 2.3 DNA extraction, PCR amplification, and library construction

Total DNA was extracted through mechanical disruption and a phenolic extraction protocol (Stevenson and Weimer, 2007), with a modification of the PCSA method (phenol:chloroform with bead beating II), as previously described (Henderson et al., 2013), which have proven effective for obtaining high-yield DNA that accurately represents ruminal microbial communities.

Briefly, sample preparation for DNA extraction was initiated using a stomacher apparatus (Seward Stomacher 400; Seward Inc., West Sussex, UK), which employs dual paddles to mechanically agitate samples and detach fiber-associated microbial cells from solid particles while simultaneously fragmenting them into smaller components. Samples (10 mL by volume) was combined with 40 mL of 1 × Phosphate-Buffered Saline (PBS) within stomacher bags with 0.5 mm filter insert (BA6040/CLR/STR; Seward Inc.). After 5 min, the corner of the bag was cut, and the liquid was removed. The resulting buffer suspension underwent centrifugation at 5,000 × g for 1 h to concentrate the cells. Subsequently, the supernatant was discarded, and the resulting pellet was reconstituted in 5 mL of PBS. Then, samples underwent treatment with extraction buffer containing Tris–HCl (0.1 M), EDTA (0.01 M), and NaCl (0.15 M). The extraction buffer served as a negative control throughout the DNA isolation procedure. Duplicate aliquots of 1 mL from the reconstituted sample were transferred to microcentrifuge tubes containing 700 μL of equilibrated phenol (Sigma-Aldrich, St. Louis, MO), 50 μL of 20% sodium dodecyl sulfate solution (Sigma-Aldrich, St. Louis, MO), and 0.5 g of zirconium/silica beads (0.1 mm diameter, BioSpec Products, Bartlesville, OK). Cellular disruption was achieved through bead-beating methodology, conducted in two 2-min intervals with an intermediate 10-min incubation at 60 °C between beating cycles. Following mechanical lysis, tubes were subjected to centrifugation at 14,000 × g for 10 min, and DNA recovery was performed from 850 μL of the resulting supernatant. DNA purification was accomplished through sequential vortexing of the supernatant with equivalent volumes of phenol-chloroform-isoamyl alcohol [PCI, 25:24:1 (vol/vol/vol)], followed by centrifugation at 14,000 × g for 10 min. DNA precipitation was achieved by gentle mixing of 500 μL supernatant with 50 μL of 2 M sodium acetate and 300 μL isopropyl alcohol. The resulting mixture underwent incubation for 2 h or overnight at −20 °C, followed by centrifugation at 14,000 × g for 20 min at 4 °C. The final step involved washing the pellet with 1 mL of ice-cold 70% ethanol and air-drying for 90 min or overnight before reconstitution in elution buffer (10 mM Tris and 1 mM EDTA).

DNA concentrations were determined using Qubit fluorometer reagents (Invitrogen, Waltham, MA). Amplification of the 16S rRNA gene V4 hypervariable region was conducted through polymerase chain reaction (PCR) employing universal bacterial primers (515F: 5’-GTGCCAGCMGCCGCGGTAA-3′; 806R: 5’-GGACTACHVGGGTWTCTAAT-3′), according to Kozich et al. (2013). The primer sequences incorporated adapters designed for compatibility with Illumina sequencing technology (F- AATGATACGGCGACCACCGAGATCTACAC; R- CAAGCAGAAGACGGCATACGAGAT) and contained distinct barcoding sequences to enable sample multiplexing: forward primers carried 16 distinct 8-bp barcodes, while reverse primers contained 24 distinct 8-bp barcodes.

PCR amplification reactions were assembled by combining 25–50 ng of template DNA with 0.2 μmol/L of each primer in a final reaction volume of 25 μL containing 2 × KAPA HiFi HotStart ReadyMix (KAPA Biosystems, Wilmington, MA). Thermal cycling was performed using a Bio-Rad S1000 thermocycler (Bio-Rad Laboratories, Hercules, CA, USA) under the following parameters: initial denaturation at 95 °C for 3 min, followed by 25 amplification cycles consisting of 95 °C for 30 s, 55 °C for 30 s, and 72 °C for 30 s, with a final extension step at 72 °C for 5 min.

Amplification products were analyzed by electrophoresis on 1% (w/v) low-melting-point agarose gels prepared with AquaPor low-melt agarose (National Diagnostics, Atlanta, GA) and visualized using SYBRSafe DNA gel stain (Invitrogen, Waltham, CA). Successful amplification was confirmed by the presence of bands at approximately 380 bp. Target bands were excised from gels, recovered, and purified using the Zymoclean Gel DNA Recovery Kit (Zymo Research, Irving, CA). No-template negative controls were incorporated into each PCR batch, and any detection of amplification in negative controls resulted in complete re-processing of all samples in that batch beginning from PCR setup. For negative controls showing no amplification, the corresponding gel region (~380 bp) was excised and processed for sequencing to provide additional confirmation of contamination absence.

Purified DNA from gel extraction was quantified using a Qubit fluorometer and 96-well plate spectrophotometer. Library construction involved creating a 4 nmol/L equimolar pool of all PCR products. The resulting library underwent sequencing on an Illumina MiSeq platform following standard Illumina protocols, utilizing a MiSeq v2 2 × 250 sequencing kit at 10 pmol/L concentration with 10% PhiX control. All sequences associated with this study were deposited into the National Center for Biotechnological Information’s Sequence Read Archive and is available under BioProject accession number PRJNA1307616.

### 2.4 Sequence processing and analysis

Raw fastq files generated by the sequencer underwent quality control and bioinformatics analysis using mothur v1.39.0 (Schloss et al., 2009). Initial sequence screening was performed (maxambig = 0, maxhomop = 8, minlength = 200, maxlength = 500), followed by grouping of identical sequences using the unique.seqs command. Sequences were subsequently aligned (align.seqs) against the SILVA 16S rRNA gene reference alignment database (Release 132, Pruesse et al., 2007) and subjected to additional screening to retain only sequences aligned to the target region (screen.seqs, start = 13,862, end = 23,444). Following alignment, sequences underwent filtering (filter.seqs) and re-grouping of identical sequences (unique.seqs). Highly similar sequences were consolidated using the pre.cluster command (diffs = 2), while chimeric sequences were identified and eliminated using chimera.uchime and remove.seqs commands, respectively. Taxonomic assignment was conducted using the SILVA database, with subsequent removal of non-bacterial sequences (those unclassified at the Kingdom level, Archaea, Eukaryota, cyanobacteria, and mitochondria).

Singleton sequences (appearing only once in the dataset) were eliminated (split.abund) to reduce bias from sequencing errors, and uncorrected pairwise distances between sequences were computed (dist.seqs). Sequence clustering into operational taxonomic units (OTUs) was performed using cluster.split (method = opti, cutoff = 0.03) at 97% sequence similarity threshold. Sample coverage was assessed using Good’s index (Good, 1953), while OTU taxonomic classification was determined using the GreenGenes database (August 2013 release) (DeSantis et al., 2006). Sample normalization was conducted by subsampling to the minimum sequence count observed across all samples, which corresponded to 9,100 sequences. A 0.1% total abundance threshold was implemented on the normalized dataset using filter.shared (minpercent = 0.001). Community structure analysis was subsequently performed using non-metric multidimensional scaling (NMDS) based on Bray–Curtis dissimilarity index (Bray and Curtis, 1957).

Microbial network analysis was performed to identify co-occurrence patterns among bacterial genera. To investigate the structure of microbial interactions within the ruminal and cecal compartments, co-occurrence networks were constructed at the genus level for both the Control and Narasin treatment groups. Spearman’s rank correlation coefficients (*ρ*) were calculated for all pairwise comparisons of genera. Significant correlations, defined as those with an absolute coefficient |ρ| > 0.3 and a *p*-value < 0.05, were used to construct the networks. Network visualization and analysis were performed using the igraph package in R. In the resulting graphs, nodes represent genera and edges represent significant correlations. Nodes were sized proportionally to the mean relative abundance of each genus, and colored according to their respective phylum. Edges were colored to represent positive (green) or negative (red) correlations. Finally, network centrality metrics (degree, closeness, and betweenness) were calculated for each node to characterize its topological importance within the microbial community.

### 2.5 Statistical analysis

All statistical analyses were performed in R version 4.3.3 (R Core Team, 2025). Prior to statistical analysis, all data were tested for normality using the Shapiro–Wilk test and homogeneity of variances using Levene’s test. Bacterial sequences were grouped into operational taxonomic units (OTUs) with 97% sequence similarity. The coverage of Good (1953) was calculated in Mothur (v.1.48.1) for all samples. All samples achieved Good’s coverage ≥93% (mean = 0.94). The OTU counts were normalized to 10,000 sequences per sample, and these normalized OTU counts were used for further analysis.

Alpha diversity was assessed using both Chao’s Richness estimate (Chao, 1984) and Shannon’s Diversity Index (Shannon, 2001), a measure of richness and evenness. Alpha diversity metrics were calculated from normalized OTU count data using the vegan package v.2.6–10 in R v.4.3.3. Shannon’s diversity index was computed using the diversity() function, while species richness was estimated using the Chao1 estimator. Statistical comparisons between treatment groups were performed separately for each compartment using Student’s t-test. Statistical significance was declared at *p* ≤ 0.05.

Beta diversity was assessed using Bray-Curtis (Bray and Curtis, 1957) and Jaccard (Jaccard, 1912) dissimilarity matrices calculated with vegdist(). Non-metric multidimensional scaling (NMDS) ordination was performed using metaMDS() with k = 2 dimensions and trymax = 100 iterations. Statistical significance of treatment effects was evaluated using PERMANOVA implemented through adonis2(). Ordination plots display 95% confidence ellipses and stress values for interpretation quality.

Taxonomic composition analysis was performed at the phylum level using phyloseq package (McMurdie and Holmes, 2013). The seven most abundant phyla were selected using taxa_sums(). For visualization, samples were grouped by treatment using merge_samples(), followed by relative abundance calculation using transform_sample_counts(). Phyla <1% abundance were grouped as ‘<1% abundance’. Statistical differences in phylum-level abundances between treatment groups were assessed using the Wilcoxon rank-sum test (Mann–Whitney U test) for each individual phylum, comparing control versus narasin-treated animals. *p*-values were adjusted for multiple comparisons using the false discovery rate (FDR) method (Benjamini and Hochberg, 1995). Phyla with FDR-adjusted *p*-values < 0.05 were considered significantly different between treatments.

## 3 Results

**Figure 1:**
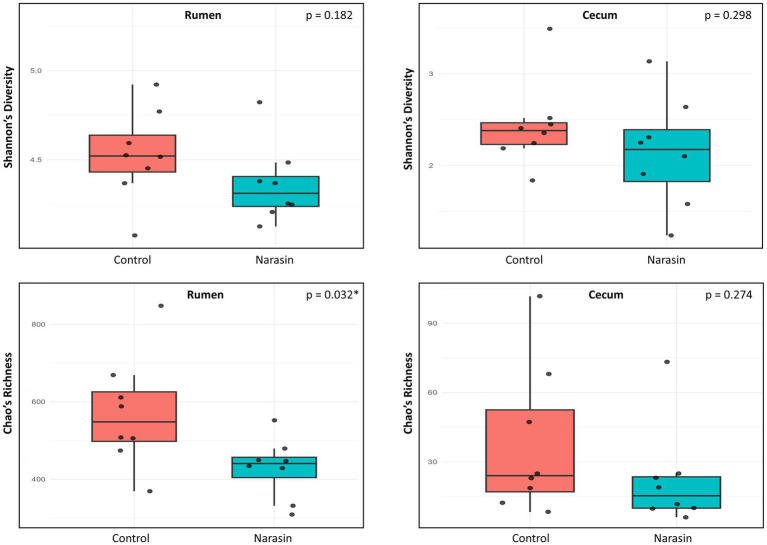
Shannon’s diversity index and Chao richness index of rumen and cecum bacteria from Nellore cattle fed narasin.

The results for the Shannon diversity index and Chao species richness are presented in Figure 1. Overall, the rumen demonstrated greater diversity and richness compared to the cecum, regardless of treatment. No differences were observed in the Shannon index for either the rumen (*p* = 0.182) or the cecum (*p* = 0.298). However, narasin reduced Chao richness only in the rumen (*p* = 0.032), not in the cecum (*p* = 0.274).

**Figure 2:**
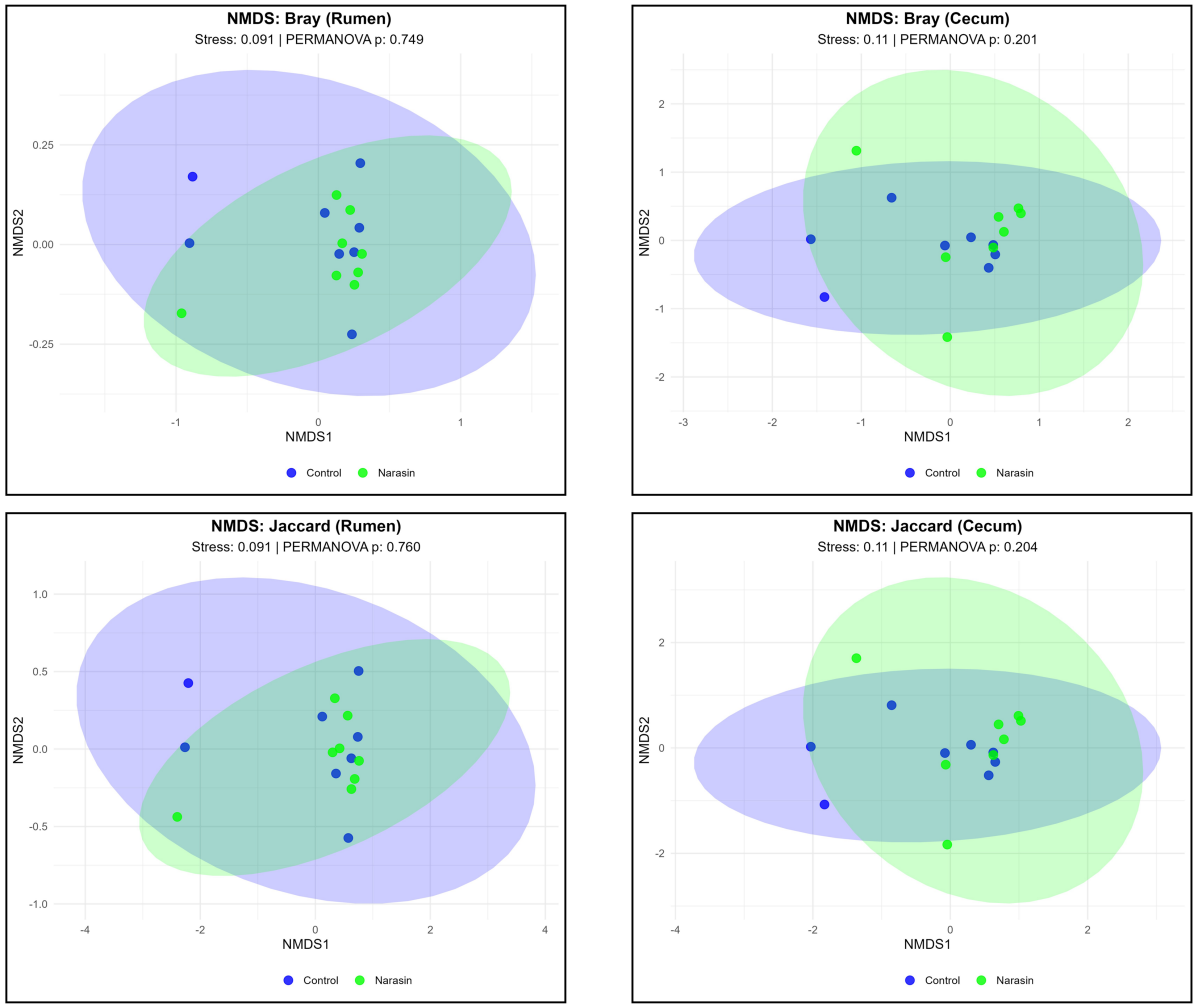
Difference of beta diversity in the two groups using principal coordinate analysis (PCoA) based on Bray-Curtis distance and Jaccard in rumen and cecum content from Nellore cattle fed narasin.

Beta-diversity analysis using NMDS ordination revealed no significant differences in overall microbial community structure between control and narasin-treated groups (*p* > 0.201), either the rumen or the cecum (Figure 2). The ordination provided reliable representation of community patterns (stress values: 0.091–0.11), indicating that while individual taxa may be affected by narasin treatment, the overall community structure remained stable. These results suggest that narasin exerts selective effects on specific bacterial taxa rather than causing broad-scale community disruption.

**Figure 3:**
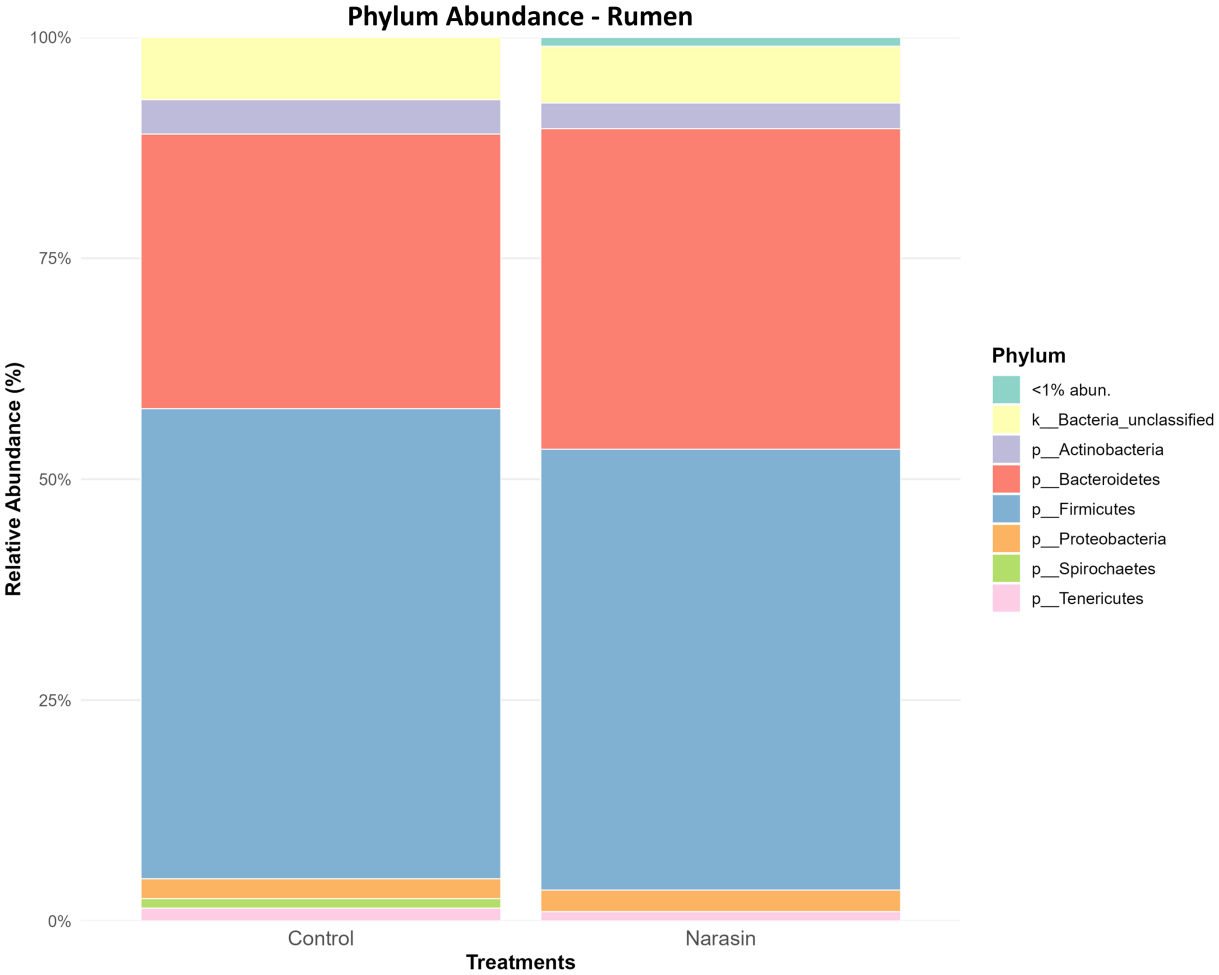
Relative abundance of rumen bacteria, at phylum level, in Nellore cattle fed narasin. Values bearing different superscripts (a, b) in a row, differ significantly (*p* = 0.05).

**Figure 4:**
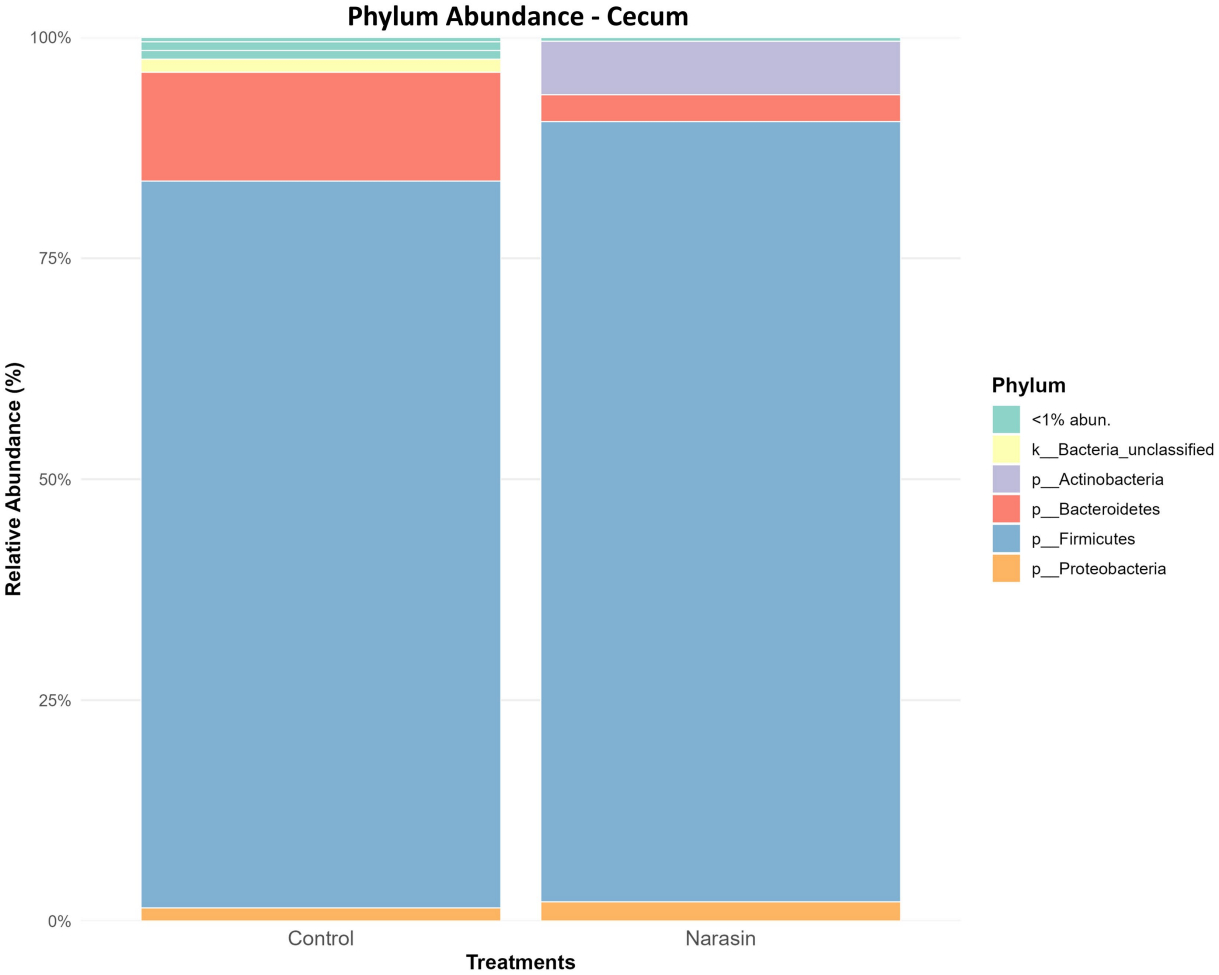
Relative abundance of cecum bacteria, at phylum level, in Nellore cattle fed narasin. Values bearing different superscripts (a, b) in a row, differ significantly (*p* = 0.05).

**Figure 5:**
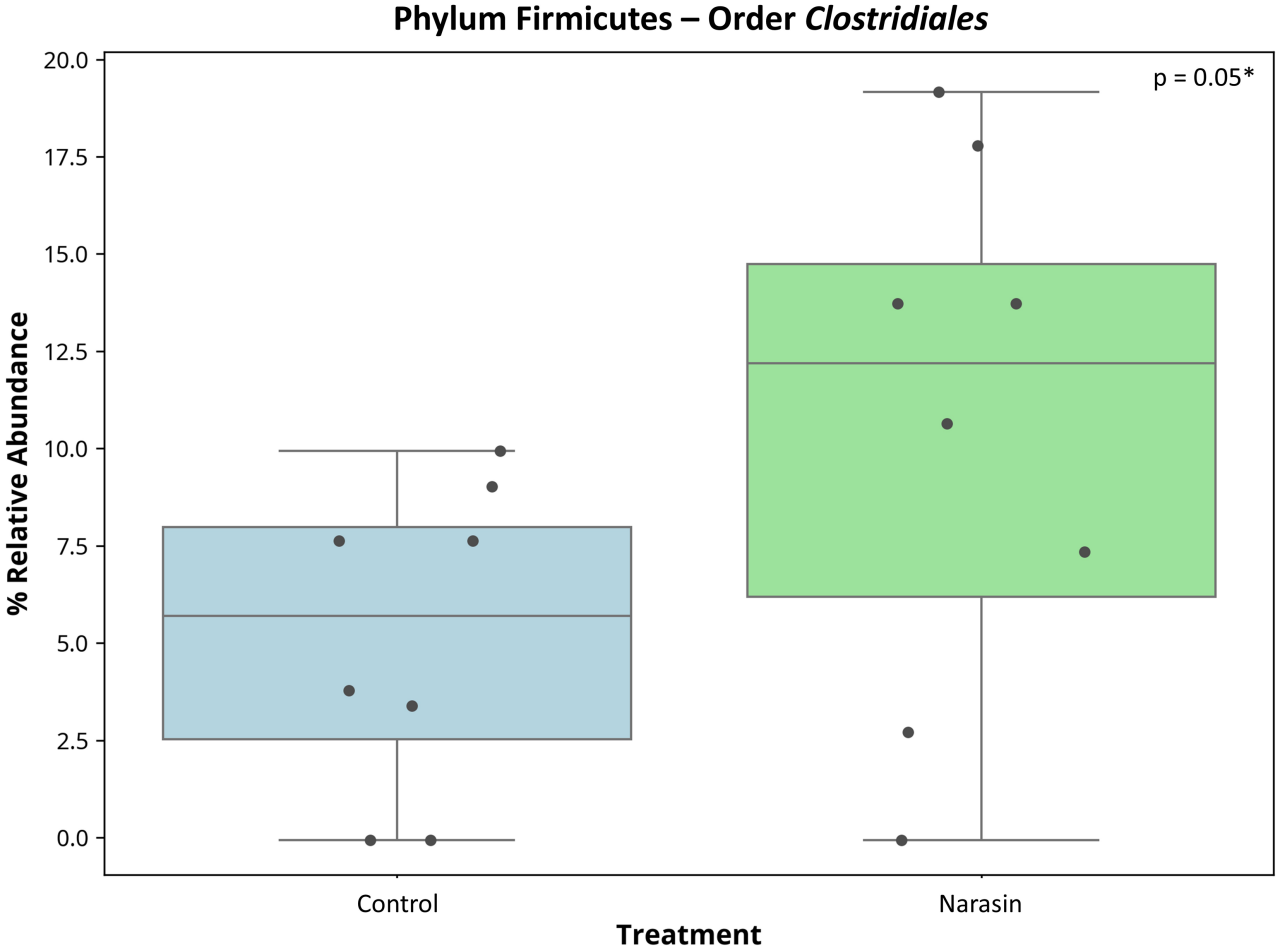
Relative abundance of cecum bacteria (*Clostridiales*) in Nellore cattle fed narasin.

**Figure 6:**
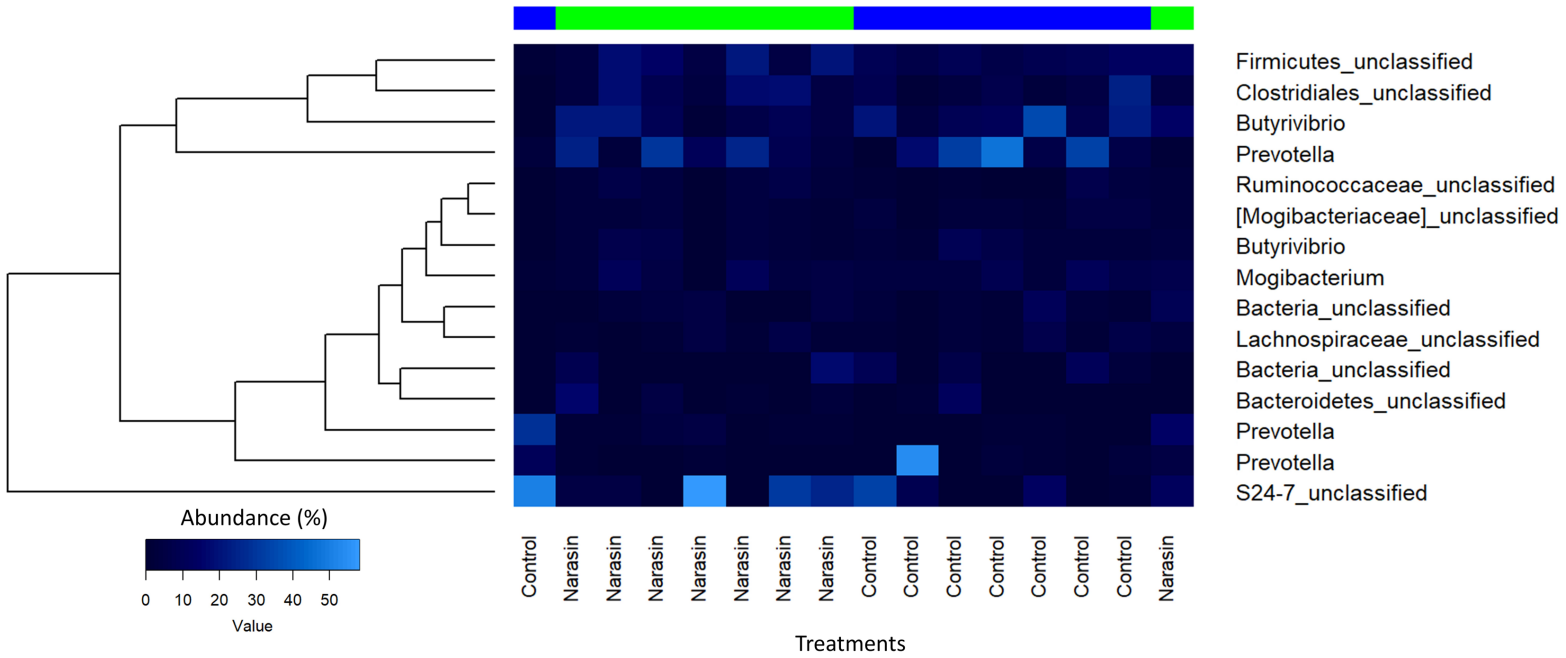
Heatmap of microbial abundance showing the main genera in the rumen of feedlot Nellore cattle fed narasin.

**Figure 7:**
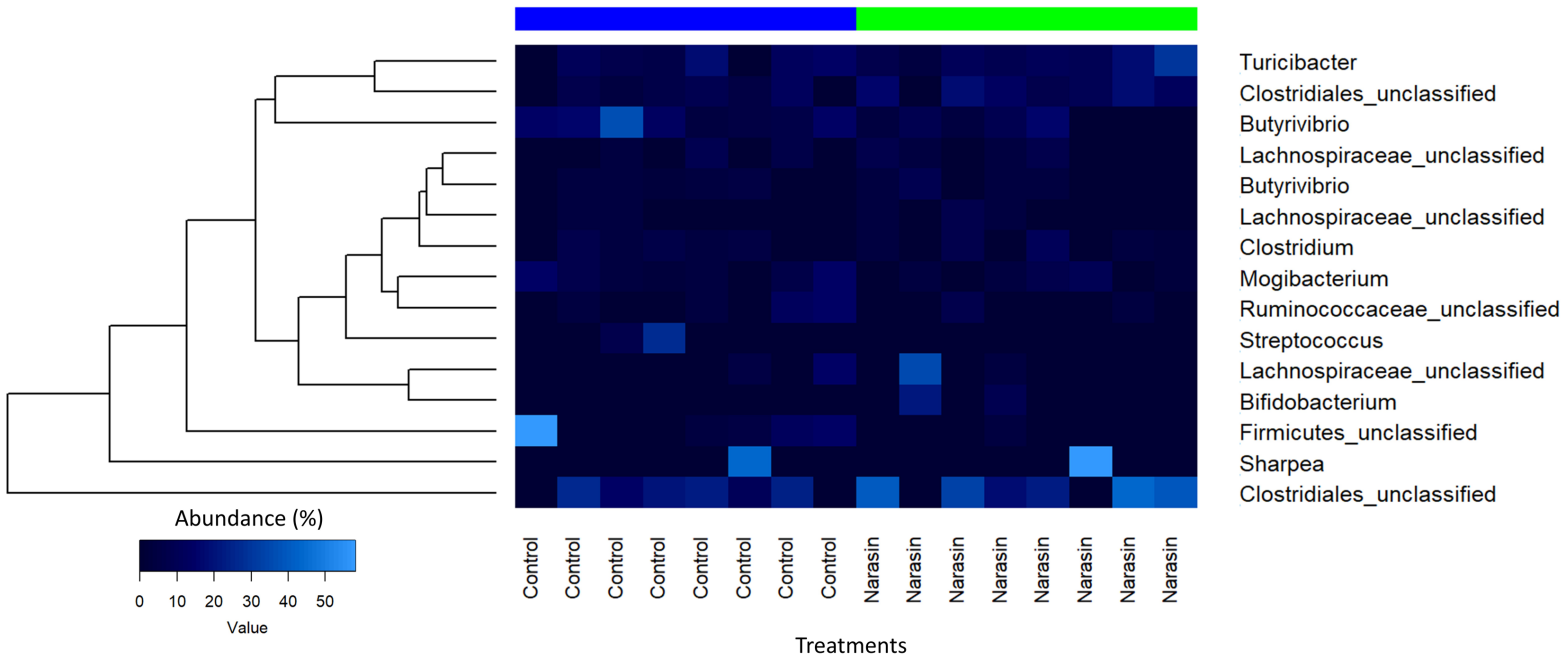
Heatmap of microbial abundance showing the main genera in the cecum of feedlot Nellore cattle fed narasin.

Regarding relative abundance in the rumen (Figure 3), there was a reduction in the abundance of the phylum *Firmicutes*, accompanied by an increase in *Bacteroidetes* in cattle-fed narasin (*p* = 0.05). Regarding relative abundance in the cecum (Figure 4), the phylum *Firmicutes* was predominant in the cecum, contrasting sharply with the rumen environment. Furthermore, there was a significant effect (*p* = 0.05) of narasin on the cecal microbiota abundance, with supplementation increasing the abundance of *Firmicutes*, particularly within the *Clostridiales* order (Figure 5). The heat map of microbial abundance for the main genera in the rumen and cecum is shown in Figures 6, 7, respectively. No significant differences due to narasin were observed in the rumen and cecum (*p* > 0.05).

**Figure 8:**
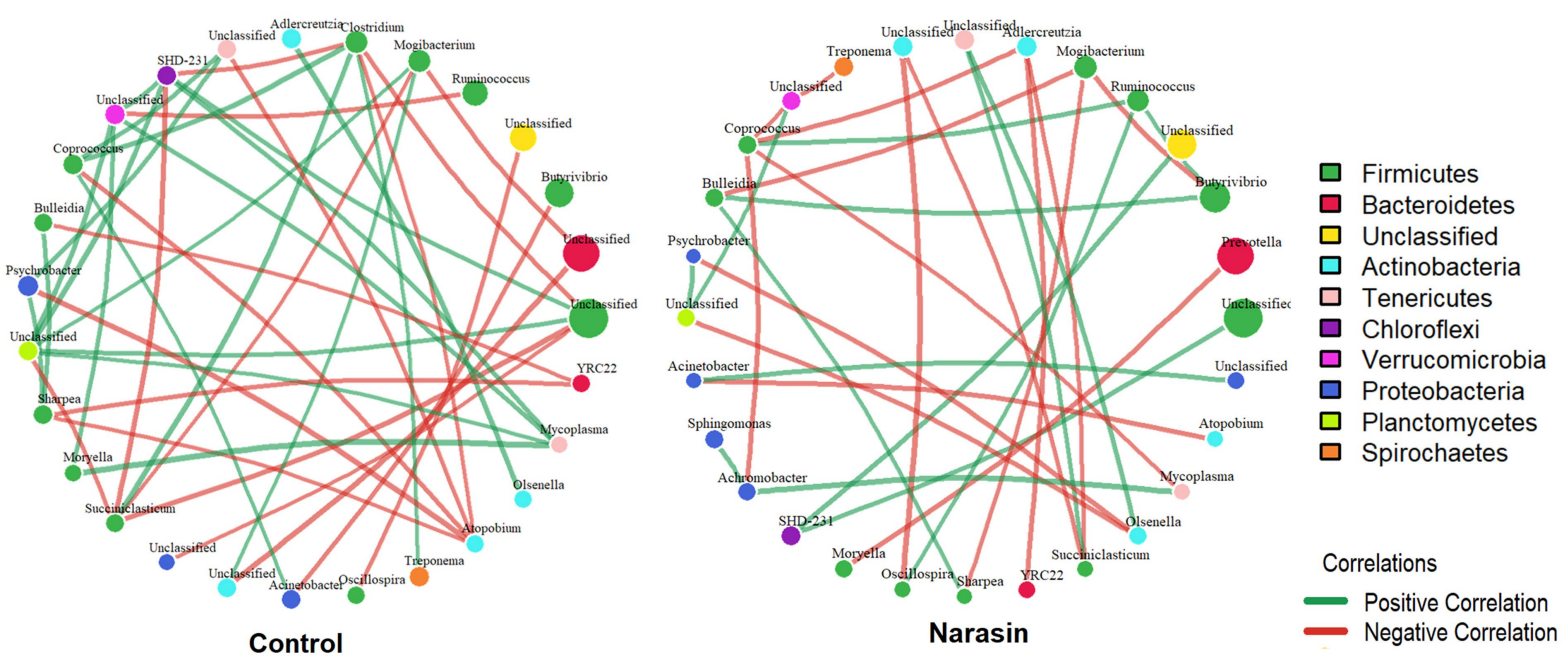
Co-occurrence networks of the ruminal microbiota in control and narasin-treated animals. Genus-level interaction networks based on 16S rRNA gene sequence data, displaying statistically significant correlations (*p* < 0.05) with an absolute Spearman’s |*ρ*| > 0.3. Each node represents a bacterial genus, with node size scaled according to its mean relative abundance within each treatment group. Node color indicates the phylum. An edge represents a significant correlation between two genera: green edges indicate a positive correlation, and red edges indicate a negative correlation. Control network – 40 correlations (52.5% positive) 27 genera from 10 phyla; narasin network – 30 correlations (46.7% positive) 28 genera from 10 phyla.

To investigate the impact of narasin on ruminal microbial interactions, we constructed genus-level co-occurrence networks for the Control and Narasin groups (Figure 8). The network from the Control group displayed greater complexity, featuring a total of 40 significant correlations, of which 21 (52.5%) were positive and 19 (47.5%) were negative. This network comprised 27 distinct genera, representing 10 different phyla. In contrast, narasin supplementation resulted in a more simplified interaction network. The Narasin group network exhibited only 30 significant correlations, a 25% reduction in the total number of connections compared to the Control group. In this network, a slight predominance of negative interactions was observed, with 16 correlations (53.3%) being negative and 14 (46.7%) positive. Despite the lower connectivity, the diversity of genera (28) and phyla (10) involved in the network was similar to that of the Control group.

**Figure 9:**
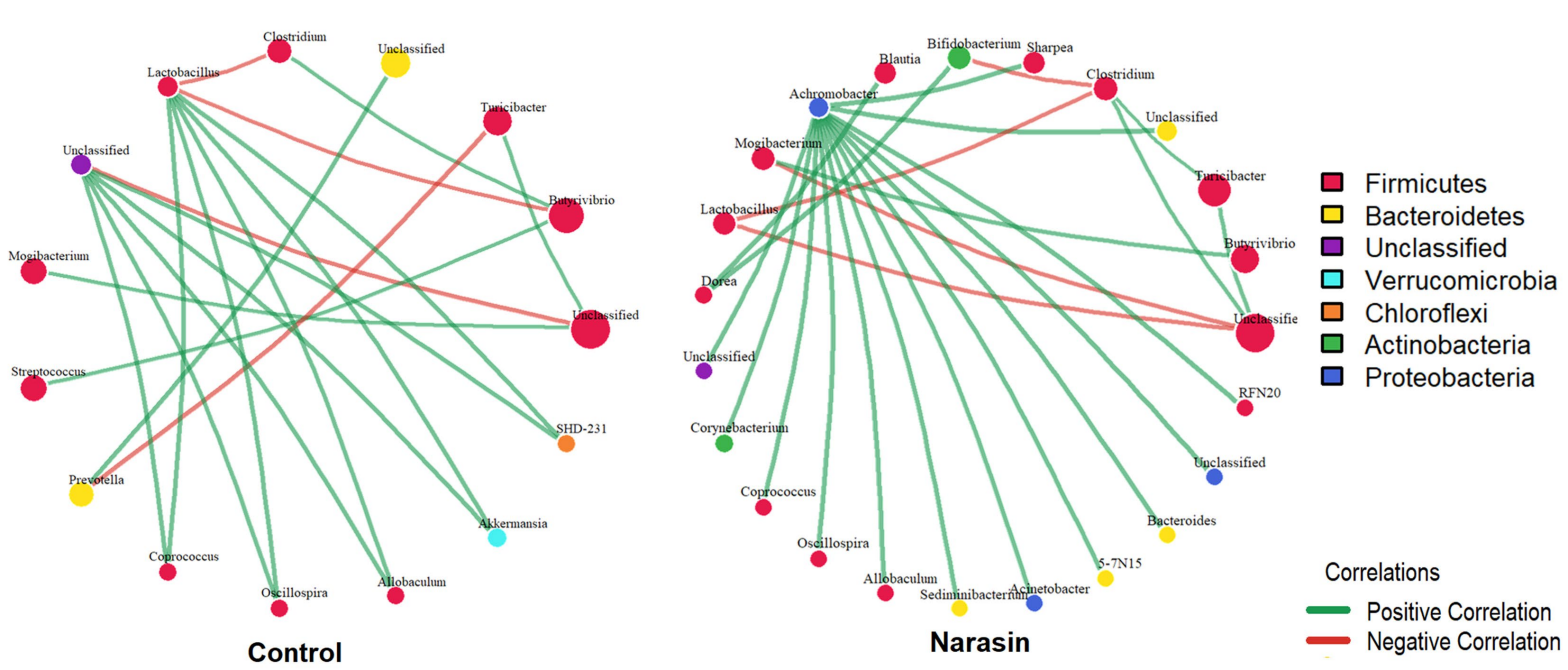
Co-occurrence networks of the cecal microbiota in Control and Narasin-treated animals. Genus-level interaction networks based on 16S rRNA gene sequence data, displaying statistically significant correlations (*p* < 0.05) with an absolute Spearman’s |ρ| > 0.3. Each node represents a bacterial genus, with node size scaled according to its mean relative abundance within each treatment group. Node color indicates the phylum. An edge represents a significant correlation between two genera: green edges indicate a positive correlation, and red edges indicate a negative correlation. The networks for the Control (left) and Narasin (right) groups are shown, illustrating the dramatic increase in network density and the shift to an exclusively positive interaction structure following narasin supplementation. Control Network – 19 correlations (78.9% positive) 15 genera from 5 phyla; Narasin Network – 23 correlations (82.6% positive) 23 genera from 5 phyla.

Regarding the cecum, the network of the Control group was characterized by 19 significant correlations among 15 genera from 5 different phyla (Figure 9). This network was dominated by positive interactions, which accounted for 15 of the correlations (78.9%), while 4 were negative (21.1%). In contrast, the cecal network in the Narasin-treated group showed a modest but notable increase in connectivity, with 23 significant correlations among 23 genera from 5 phyla. This network exhibited an even stronger positive bias, with 19 positive correlations (82.6%) and 4 negative correlations (17.4%).

## 4 Discussion

The cecum plays a crucial role in post-ruminal fermentation, yet it remains an underexplored aspect of beef cattle microbiome research. Most studies focus on the rumen because of its primary function in fiber degradation and fermentation (Henderson et al., 2015). However, the cecum, although smaller in volume, can still influence overall digestive efficiency and microbial metabolite production (Li et al., 2019). Alterations in cecal microbiota composition may have physiological implications, potentially affecting nutrient absorption, hindgut fermentation, and even rumen function through enterohepatic circulation (Malmuthuge and Guan, 2017). Thus, understanding the impact of dietary additives such as narasin on the cecum is essential for a comprehensive evaluation of their effects on ruminant digestion and health.

Given the well-established selective mode of action of ionophores, the absence of broad-scale shifts in microbial diversity was expected, with narasin primarily exerting targeted effects on specific bacterial populations. The lack of significant differences in the Shannon diversity index suggests that narasin did not affect the evenness of the microbial communities in either the rumen or the cecum. This aligns with findings from earlier research indicating that ionophores typically modify specific bacterial populations rather than cause widespread diversity shifts (Russell and Strobel, 1989; McCann et al., 2017). However, the observed reduction in Chao richness in the rumen indicates a decrease in species diversity, which aligns with the known inhibitory effects of ionophores on Gram-positive bacteria (Callaway et al., 2003). This reduction is likely driven by the selective suppression of *Firmicutes*, a major bacterial group in the rumen, which comprises key fiber-digesting species (Russell and Houlihan, 2003). Moreover, narasin did not significantly affect beta-diversity in either the rumen or the cecum. The NMDS ordination provided reliable representation of community patterns (stress values: 0.091–0.11), indicating that while individual taxa may be affected by narasin treatment, the overall community structure remained stable. These results suggest that narasin exerts selective effects on specific bacterial taxa rather than causing broad-scale community disruption, consistent with the known selectivity of ionophores toward Gram-positive bacteria (Russell and Strobel, 1989; Russell and Houlihan, 2003; Marques and Cooke, 2021).

The decrease in *Firmicutes* and the increase in *Bacteroidetes* in the rumen of cattle-fed narasin is consistent with the mode of action of ionophores, which preferentially target Gram-positive bacteria by disrupting their ion gradients (Russell and Houlihan, 2003). Given that narasin’s mechanism of action is similar to monensin, a higher relative abundance of *Bacteroidetes* (Gram-negative) and a lower relative abundance of *Firmicutes* (Gram-positive) are expected in steers fed narasin. This shift is anticipated, considering that *Bacteroidetes* are Gram-negative and generally more resistant to ionophores (Mao et al., 2016). However, the absence of significant differences in other phyla suggests that functional redundancy within the microbial community may compensate for compositional shifts, enabling the rumen microbiota to sustain its overall fermentative capacity (Henderson et al., 2015).

Interestingly, a different pattern was observed in the cecal microbiota abundance. First, the phylum *Firmicutes* dominated in the cecum, which is quite distinct from the rumen environment. This result is consistent with the fact that the rumen serves as the primary fermentation chamber for forage, so little fiber reaches the cecum for fermentation, which may explain the low abundance of *Bacteroidetes* there (Deusch et al., 2017). On the other hand, due to the rumen liquid passage rate, a significant amount of concentrate can reach the cecum and be fermented, potentially explaining the dominance of *Firmicutes* in that area (Mao et al., 2016).

Moreover, the cecum exhibited a distinct response to narasin supplementation. Unlike the rumen, where *Firmicutes* showed a reduction, the cecum demonstrated a significant increase in *Firmicutes* abundance, particularly within the *Clostridiales* order. This finding is novel, as few studies have examined the effects of ionophores on the cecal microbiota of ruminants. The predominance of *Firmicutes* in the cecum is consistent with differences in substrate availability and fermentation dynamics. While the rumen primarily ferments fiber, the cecum ferments residual starch and soluble carbohydrates that escape ruminal digestion (Deusch et al., 2017). The increase in *Clostridiales* suggests a shift toward microbial populations that may be better adapted to utilizing these substrates under ionophore influence, though functional validation would be required to confirm this hypothesis. Thus, these results demonstrate that narasin supplementation induces specific changes in the cecal microbiota, the functional significance of which warrants further investigation.

Based on existing literature, cecal microbiota modifications have the potential to extend beyond local fermentation processes. There is increasing evidence indicating that changes in hindgut microbial composition can affect systemic metabolism and immune function through microbial metabolites such as SCFA and secondary bile acids (Malmuthuge and Guan, 2017; Huws et al., 2018). Additionally, changes in the cecal microbiome could potentially affect rumen function through enterohepatic recycling of microbial metabolites and microbial cross-talk between gut compartments (Mao et al., 2016). However, this study did not assess metabolite production or functional outcomes, and future research should investigate whether shifts in cecal microbial populations align with functional changes that may have systemic implications.

Our co-occurrence network analysis revealed that narasin supplementation induced a significant restructuring of the ruminal microbial community architecture. The 25% reduction in total interactions within the narasin group (from 40 to 30 edges) suggests that the ionophore acts not only by altering the abundance of specific taxa but also by simplifying the ecosystem by reducing the interdependence among genera. This loss of connectivity is a classic indicator of an ecosystem responding to a chemical stressor, potentially leading to the destabilization of established ecological niches, affecting both synergistic (cooperative) and antagonistic (competitive) relationships (Shade et al., 2012). Such simplification of microbial networks has been previously observed in ruminal environments under dietary pressures, suggesting a shift toward a less resilient but more specialized community (Hernandez-Sanabria et al., 2012). The shift in the balance of correlations, from a slight positive dominance in the control group (52.5%) to a slight negative dominance in the narasin group (53.3%), is particularly noteworthy. This may imply that under the selective pressure of narasin, competitive or exclusionary relationships become more prevalent or detectable compared to mutualistic ones. Ionophores like narasin selectively target Gram-positive bacteria by disrupting their transmembrane ion gradients, an action that can open ecological niches for more resistant microorganisms to establish new competitive relationships for substrates (Russell and Strobel, 1989; Callaway et al., 2003). Analyzing specific interactions highlights these dynamics. In the Control group, the strong positive correlation (r = 0.90) between *Clostridium* and *Coprococcus*, both members of the *Firmicutes* phylum, suggests a cooperative relationship, likely linked to fiber degradation and butyrate production (Pryde et al., 2002). This specific interaction was absent in the narasin network, indicating that narasin may have disrupted this synergy.

In the cecum, the microbial interaction network of the narasin-treated group was different from that observed in the rumen. Rather than simplifying the network, oral narasin supplementation was associated with a moderate increase in microbial connectivity and a shift toward more cooperative interactions. The increase in total interactions (from 19 to 23) and the enhanced positive correlation structure (78.9 to 82.6% positive) indicate the establishment of a more cooperative and potentially more stable community (Coyte et al., 2015). We hypothesize that this is an indirect effect, driven by the primary action of the ionophore in the upper gastrointestinal tract. By altering ruminal fermentation, narasin likely modifies the composition of digesta flowing into the cecum (Duffield et al., 2012). This ruminal outflow, containing different microbial cells, metabolites, and fermentation byproducts, would create a new ecological foundation for the hindgut community. This altered environment appears to have selected for a highly specialized consortium of bacteria, which is consistent with our finding that narasin supplementation increased the abundance of *Firmicutes*, particularly within the *Clostridiales* order. Strong positive correlations were maintained among key cecal fermenters, including *Coprococcus*, *Oscillospira*, and other members of the *Clostridiales* order were observed. This pattern suggests that narasin selectively modulates specific microbial interactions rather than uniformly promoting cooperation, resulting in a more structured and potentially more efficient cecal ecosystem (Berry and Widder, 2014). Moreover, the narasin treatment was associated with the emergence of specific competitive interactions, particularly negative correlations involving *Clostridium* with both *Lactobacillus* and *Bifidobacterium*, suggesting altered competitive dynamics within the *Firmicutes* phylum. This finding is consistent with well-documented antagonistic relationships between these genera in the intestinal environment. *Bifidobacterium* species have been shown to exert direct inhibitory effects against *Clostridium* through multiple mechanisms, including acidification of the intestinal environment, competition for nutrients and adhesion sites, and production of antimicrobial compounds (Wei et al., 2018). Similarly, *Lactobacillus* strains demonstrate competitive exclusion against various *Clostridium* species, particularly through the production of organic acids and bacteriocins that create unfavorable conditions for clostridial growth (Li et al., 2017). This difference in network complexity between rumen and cecum likely reflects the distinct functional roles of these compartments, with the rumen serving as the primary fermentative chamber processing diverse dietary components, while the cecum represents a more specialized environment focused on secondary fermentation (Wang et al., 2017; Mi et al., 2018).

The present findings contribute to the limited body of literature on cecal microbiota in ruminants and highlight the necessity for further investigation into its role in digestive physiology. To establish causal relationships between taxonomic shifts and functional outcomes, future studies should incorporate metabolomic and transcriptomic approaches to clarify the functional consequences of microbial shifts induced by dietary interventions such as narasin. Understanding the full extent of microbial interactions across gut compartments is crucial for optimizing feed strategies that enhance both rumen efficiency and overall animal health.

## 5 Conclusion

Narasin appears to exert a selective effect on specific bacterial populations, such as *Firmicutes*, particularly within the *Clostridiales* order, without significantly altering overall microbial diversity. This effect may be linked to reduced richness in the rumen and shifts in the cecal microbiota, potentially impacting feed efficiency by favoring certain fermentation pathways. Moreover, our results demonstrate that narasin remodels the ruminal microbial ecology by reducing the complexity of inter-genus interactions. This network reconfiguration, characterized by fewer connections and a shift toward negative correlations, represents a fundamental mechanism explaining the ionophore’s effects on the rumen environment. However, further studies are necessary to understand the functional implications of these microbial shifts and how they translate into production benefits.

## Data Availability

The data presented in this study are publicly available. The data can be found at: https://www.ncbi.nlm.nih.gov, PRJNA1307616.

## References

[ref9001] Benjamini Y. Hochberg Y. (1995). Controlling the false discovery rate: A practical and powerful approach to multiple testing. Journal of the Royal Statistical Society: Series B, 57, 289–300. doi: 10.1111/j.2517-6161.1995.tb02031.x

[ref1] Berry D. Widder S. (2014). Deciphering microbial interactions and detecting keystone species with co-occurrence networks. Front. Microbiol. 5:219. doi: 10.3389/fmicb.2014.00219, 24904535 PMC4033041

[ref2] Bray J. R. Curtis J. T. (1957). An ordination of the upland forest communities of southern Wisconsin. Ecol. Monogr. 27, 325–349. doi: 10.2307/1942268

[ref9002] Brown M. S. Ponce C. H. Pulikanti R. (2006). Adaptation of beef cattle to high-concentrate diets: Performance and ruminal metabolism. Journal of Animal Science, 84, E25–E33. doi: 10.2527/2006.8413_supplE25x 16582090

[ref3] Callaway T. R. Edrington T. S. Rychlik J. L. Nisbet D. J. (2003). Ionophores: their use as ruminant growth promotants and impact on food safety. Curr. Issues Intest. Microbiol. 4, 43–51. 14503688

[ref9003] Chao A. (1984). Nonparametric estimation of the number of classes in a population. Scandinavian Journal of Statistics, 11, 265–270.

[ref4] Coyte K. Z. Schluter J. Foster K. R. (2015). The ecology of the microbiome: networks, competition, and stability. Science 350, 663–666. doi: 10.1126/science.aad2602, 26542567

[ref5] DeSantis T. Z. Hugenholtz P. Larsen N. Rojas M. Brodie E. L. Keller K. . (2006). Greengenes, a chimera-checked 16S rRNA gene database and workbench compatible with ARB. Appl. Environ. Microbiol. 72, 5069–5072. doi: 10.1128/AEM.03006-05, 16820507 PMC1489311

[ref6] Deusch S. Camarinha-Silva A. Conrad J. Beifuss U. Rodehutscord M. Seifert J. (2017). A structural and functional elucidation of the rumen microbiome influenced by various diets and microbial networking. Front. Microbiol. 8:1605. doi: 10.3389/fmicb.2017.01605 28883813 PMC5573736

[ref8] Duffield T. F. Merrill J. K. Bagg R. N. (2012). Meta-analysis of the effects of monensin in beef cattle on feed efficiency, body weight gain, and dry matter intake. J. Anim. Sci. 90, 4583–4592. doi: 10.2527/jas.2011-4812, 22859759

[ref9] Fox D. G. Tedeschi L. O. Tylutki T. P. (2004). The ruminant nutrition system: An applied model for predicting nutrient requirements and feed utilization in ruminants. Ithaca, NY: Cornell University.

[ref10] Golder H. M. Lean I. J. (2024). Ruminal acidosis and its definition: a critical review. J. Dairy Sci. 107, 10066–10098. doi: 10.3168/jds.2024-24817, 39218070

[ref11] Good I. J. (1953). The population frequencies of species and the estimation of population parameters. Biometrika 40, 237–264. doi: 10.1093/biomet/40.3-4.237

[ref12] Henderson G. Cox F. Ganesh S. Jonker A. Young W. Janssen P. H. (2015). Rumen microbial community composition varies with diet and host, but a core microbiome is found across a wide geographical range. Sci. Rep. 5:14567. doi: 10.1038/srep14567, 26449758 PMC4598811

[ref13] Henderson G. Cox F. Kittelmann S. Miri V. H. Zethof M. Noel S. J. . (2013). Effect of DNA extraction methods and sampling techniques on the apparent structure of cow and sheep rumen microbial communities. PLoS One 8:e74787. doi: 10.1371/journal.pone.0074787, 24040342 PMC3770609

[ref14] Hernandez-Sanabria E. Goonewardene L. A. Wang Z. Durunna O. N. Moore S. S. Guan L. L. (2012). Impact of feed efficiency and diet on adaptive variations in the bacterial community in the rumen fluid of cattle. Appl. Environ. Microbiol. 78, 1203–1214. doi: 10.1128/AEM.05114-11 22156428 PMC3273029

[ref15] Huws S. A. Creevey C. J. Oyama L. B. Mizrahi I. Denman S. E. Popova M. . (2018). Addressing global ruminant agricultural challenges through understanding the rumen microbiome: past, present, and future. Front. Microbiol. 9:2161. doi: 10.3389/fmicb.2018.02161, 30319557 PMC6167468

[ref16] Jaccard P. (1912). The distribution of the flora in the alpine zone. New Phytol. 11, 37–50. doi: 10.1111/j.1469-8137.1912.tb05611.x

[ref17] Kozich J. J. Westcott S. L. Baxter N. T. Highlander S. K. Schloss P. D. (2013). Development of a dual-index sequencing strategy and curation pipeline for analyzing amplicon sequence data on the MiSeq Illumina sequencing platform. Appl. Environ. Microbiol. 79, 5112–5120. doi: 10.1128/AEM.01043-13, 23793624 PMC3753973

[ref18] Li F. Hitch T. C. A. Chen Y. Creevey C. J. Guan L. L. (2019). Comparative metagenomic and metatranscriptomic analyses reveal the response of rumen microbiota to nutrient availability. Microbiome 7:6. doi: 10.1186/s40168-019-0618-5 30642389 PMC6332916

[ref19] Li Z. Wang W. Liu D. Guo Y. (2017). Effects of *Lactobacillus acidophilus* on gut microbiota composition in broilers challenged with *Clostridium perfringens*. PLoS One 12:e0188634. doi: 10.1371/journal.pone.0188634, 29190649 PMC5708699

[ref20] Limede A. C. Polizel D. M. Silva J. T. de Almeida M. T. C. Cônsolo N. R. B. (2021). Effects of narasin on ruminal fermentation and performance of Nellore cattle. Livest. Sci. 99:skab005. doi: 10.1093/jas/skab005

[ref21] Malmuthuge N. Guan L. L. (2017). Understanding the gut microbiome of dairy calves: opportunities to improve early-life gut health. J. Dairy Sci. 100, 5996–6005. doi: 10.3168/jds.2016-12239, 28501408

[ref22] Mao S. Y. Huo W. J. Zhu W. Y. (2016). Microbiome–metabolome analysis reveals unhealthy alterations in the composition and metabolism of ruminal microbiota with increasing dietary grain in a goat model. Environ. Microbiol. 18, 525–541. doi: 10.1111/1462-2920.12724 25471302

[ref23] Marques R. S. Cooke R. F. (2021). Effects of ionophores on ruminal function of beef cattle. Animals 11:2871. doi: 10.3390/ani11102871, 34679890 PMC8532634

[ref24] McCann J. C. Elolimy A. A. Loor J. J. (2017). Rumen microbiome, probiotics, and fermentation additives. Vet. Clin. North Am. Food Anim. Pract. 33, 539–553. doi: 10.1016/j.cvfa.2017.06.009 28764865

[ref25] McMurdie P. J. Holmes S. (2013). Phyloseq: an R package for reproducible interactive analysis and graphics of microbiome census data. PLoS One 8:e61217. doi: 10.1371/journal.pone.0061217, 23630581 PMC3632530

[ref26] Mi L. Yang B. Hu X. Luo Y. Liu J. Yu Z. . (2018). Comparative analysis of the microbiota between sheep rumen and rabbit cecum provides new insight into their differential methane production. Front. Microbiol. 9:575. doi: 10.3389/fmicb.2018.00575, 29662480 PMC5890152

[ref27] Miszura A. A. Ribeiro J. M. Cooke R. F. Marques R. S. Cappellozza B. I. (2018). Effects of narasin on ruminal fermentation dynamics and plasma metabolites in beef cattle. J. Anim. Sci. 96:441. doi: 10.1093/jas/sky404.964

[ref9004] Nagaraja T. G. Titgemeyer E. C. (2007). Ruminal acidosis in beef cattle: The current microbiological and nutritional outlook. Journal of Dairy Science, 90, E17–E38. doi: 10.3168/jds.2006-478 17517750

[ref9005] Owens F. N. Secrist D. S. Hill W. J. Gill D. R. (1998). Acidosis in cattle: A review. Journal of Animal Science, 76, 275–286. doi: 10.2527/1998.761275x 9464909

[ref28] Polizel D. M. Cappellozza B. I. Hoe F. Lopes C. N. Barroso J. P. R. Pires A. V. (2020). Effects of narasin supplementation on dry matter intake and rumen fermentation characteristics of Bos indicus steers fed a high-forage diet. Transl. Anim. Aci. 4, 118–128. doi: 10.1093/tas/txz164 PMC7200564 32704972

[ref29] Pruesse E. Quast C. Knittel K. Fuchs B. M. Ludwig W. Peplies J. . (2007). SILVA: a comprehensive online resource for quality checked and aligned ribosomal RNA sequence data compatible with ARB. Nucleic Acids Res. 35, 7188–7196. doi: 10.1093/nar/gkm864, 17947321 PMC2175337

[ref30] Pryde S. E. Duncan S. H. Hold G. L. Stewart C. S. Flint H. J. (2002). The microbiology of butyrate formation in the human colon. FEMS Microbiol. Lett. 217, 133–139. doi: 10.1111/j.1574-6968.2002.tb11467.x, 12480096

[ref31] R Core Team. (2025) R: A language and environment for statistical computing. Vienna, Austria: R Foundation for Statistical Computing.

[ref32] Russell J. B. Houlihan A. J. (2003). Ionophore resistance of ruminal bacteria and its potential impact on human health. FEMS Microbiol. Rev. 27, 65–74. doi: 10.1016/S0168-6445(03)00019-6, 12697342

[ref33] Russell J. B. Strobel H. J. (1989). Effect of ionophores on ruminal fermentation. Appl. Environ. Microbiol. 55, 1–6. doi: 10.1128/aem.55.1.1-6.1989, 2650616 PMC184044

[ref34] Sardinha L. A. Cooke R. F. Marques R. S. Cappellozza B. I. Moriel P. (2020). Effects of narasin on growth performance and metabolism of yearling bulls consuming a high-grain diet. J. Anim. Sci. 4:txaa030. doi: 10.1093/tas/txaa030

[ref35] Schloss P. D. Westcott S. L. Ryabin T. Hall J. R. Hartmann M. Hollister E. B. . (2009). Introducing mothur: open-source, platform-independent, community-supported software for describing and comparing microbial communities. Appl. Environ. Microbiol. 75, 7537–7541. doi: 10.1128/AEM.01541-09, 19801464 PMC2786419

[ref36] Shade A. Peter H. Allison S. D. Baho D. L. Berga M. Bürgmann H. . (2012). Fundamentals of microbial community resistance and resilience. Front. Microbiol. 3:417. doi: 10.3389/fmicb.2012.00417 23267351 PMC3525951

[ref9006] Shannon C. E. (2001). A mathematical theory of communication. ACM SIGMOBILE Mobile Computing and Communications Review, 5, 3–55. doi: 10.1145/584091.584093

[ref37] Silva R. G. Ferraz M. V. C., Jr. Gouveia V. N. Polizel D. M. Santos M. H. Miszura A. A. . (2015). Effects of narasin in mineral mix to Nellore heifers fed with high forage. J. Anim. Sci. 93:118.

[ref9007] Silvestre A. M. Millen D. D. (2021). The 2019 Brazilian survey on nutritional practices provided by feedlot cattle consulting nutritionists. Revista Brasileira de Zootecnia, 50:e20200189. doi: 10.37496/rbz5020200189

[ref9008] Silvestre A. M. Souza J. M. Millen D. D. (2023). Adoption of adaptation protocols and feed additives to improve performance of feedlot cattle. Journal of Applied Animal Research, 51, 282–299. doi: 10.1080/09712119.2023.2191679

[ref38] Stevenson D. M. Weimer P. J. (2007). Dominance of Prevotella and low abundance of classical ruminal bacterial species in the bovine rumen revealed by relative quantification real-time PCR. Appl. Microbiol. Biotechnol. 75, 165–174. doi: 10.1007/s00253-006-0802-y, 17235560

[ref39] Wang J. Fan H. Han Y. Zhao J. Zhou Z. (2017). Characterization of the microbial communities along the gastrointestinal tract of sheep by 454 pyrosequencing analysis. Asian Australas. J. Anim. Sci. 30, 100–110. doi: 10.5713/ajas.16.0166, 27383798 PMC5205584

[ref40] Wei Y. Yang J. Wang J. Yang Y. Huang J. Gong H. . (2018). Protective effects of Bifidobacterial strains against toxigenic *Clostridium difficile*. Front. Microbiol. 9:888. doi: 10.3389/fmicb.2018.00888, 29867801 PMC5952185

